# Screening for Hepatocellular Carcinoma in Chronic Hepatitis B: An Update

**DOI:** 10.3390/v13071333

**Published:** 2021-07-10

**Authors:** James Lok, Kosh Agarwal

**Affiliations:** 1 Department of Gastroenterology, St. George’s Hospital, London SW17 0QT, UK; 2 Institute of Liver Studies, King’s College Hospital, London SE5 9RS, UK; kosh.agarwal@nhs.net

**Keywords:** chronic hepatitis B, hepatocellular carcinoma, risk score, biomarkers

## Abstract

(1) Background: Hepatocellular carcinoma (HCC) is an important cause of mortality in individuals with chronic hepatitis B infection, with screening of high-risk groups recommended in all major international guidelines. Our understanding of the risk factors involved has improved over time, encouraging researchers to develop models that predict future risk of HCC development. (2) Methods: A literature search of the PubMed database was carried out to identify studies that derive or validate models predicting HCC development in patients with chronic hepatitis B. Subsequently, a second literature search was carried out to explore the potential role of novel viral biomarkers in this field. (3) Results: To date, a total of 23 models have been developed predicting future HCC risk, of which 12 have been derived from cohorts of treatment-naïve individuals. Most models have been developed in Asian populations (*n* = 20), with a smaller number in Caucasian cohorts (n = 3). All of the models demonstrate satisfactory performance in their original derivation cohorts, but many lack external validation. In recent studies, novel viral biomarkers have demonstrated utility in predicting HCC risk in patients with chronic hepatitis B, amongst both treated and treatment-naïve patients. (4) Conclusion: Several models have been developed to predict the risk of HCC development in individuals with chronic hepatitis B infection, but many have not been externally validated outside of the Asian population. Further research is needed to refine these models and facilitate a more tailored HCC surveillance programme in the future.

## 1. Introduction

Despite the availability of a highly effective vaccine for many decades, chronic hepatitis B remains a global health challenge. Most recent estimates from the World Health Organisation suggest that over 250 million people have chronic hepatitis B infection worldwide [1], and these patients are at risk of serious complications, such as cirrhosis, hepatic decompensation, and hepatocellular carcinoma (HCC) [2,3]. Indeed, chronic infection with hepatitis B confers a 15 to 20-fold increased risk of HCC, compared with uninfected individuals [4], and potent antiviral medications such as nucleos(t)ide analogues have been shown to reduce, but not eliminate, this risk [5]. Major international guidelines, including EASL and AASLD, advocate screening in certain high-risk groups but differ in how this population of patients is defined [6,7,8]. Surveillance can be relatively costly and labour intensive, and research has been carried out over recent years to refine this process, identify composite scores that can prognosticate risk and develop a more cost-effective approach to screening. In this review, we will explore recent advances in this area, including the use of transient elastography [9,10] and novel biomarkers [11,12], and identify areas for future research.

## 2. Materials and Methods

To inform this review, articles were identified through a systematic search of the PubMed database from January 2000 until March 2021, using the search terms of “hepatitis B AND score AND hepatocellular carcinoma”. Following this, the retrieved articles were manually reviewed and included if they met the following criteria: (1) comprised untreated or treated patients with chronic hepatitis B infection; (2) reported the development and/or validation of a hepatocellular carcinoma (HCC) risk prediction score. Studies including patients with chronic hepatitis B and co-infection with human immunodeficiency virus (HIV) or hepatitis C were excluded. The Newcastle–Ottawa Scale was applied to evaluate the quality of each study, with note made of the following information: year of publication, country of study, follow-up period, results of multivariate analyses including covariates, hazard ratios and their corresponding 95% CIs. The size of the derivation and validation cohorts were recorded as well as the performance of the proposed risk models, as demonstrated by indices such as area under the receiver operating curve (AUROC), negative predictive value of the low-risk group and positive predictive value of the high-risk group. Finally, to explore the use of novel biomarkers in this field, a further search of the PubMed database was performed, using the search terms “hepatitis B AND biomarkers AND hepatocellular carcinoma”.

## 3. Role of Hepatocellular Carcinoma Screening in Chronic Hepatitis B

Primary liver cancer is a major global health problem and the third leading cause of cancer mortality worldwide. Latest data from the Global Cancer Observatory suggest that there were over 905,000 new cases of primary liver cancer worldwide in 2020, and 830,180 deaths, with only lung and colorectal cancer responsible for more cancer-related fatalities [13]. Hepatocellular carcinoma (HCC) represents about 90% of primary liver cancers and has a number of features that make it a viable target for a screening programme, including well-defined risk factors [14], a protracted subclinical course in its early stages [15], a range of different treatment modalities, including surgical options and loco-regional therapy [6], and prognosis that is strongly influenced by stage of the disease [16]. Indeed, patients with early-stage hepatocellular carcinoma have an estimated 5-year survival rate of 70–75%, compared to less than 12 months in patients with advanced disease [17]. Between 1990 and 2015, the number of new cases of liver cancer increased by 75%, driven by changing age structures and population growth [18]. The majority of cases are associated with a known underlying aetiology, most notably chronic viral infections (hepatitis B and C), alcohol excess, metabolic disorders (non-alcoholic fatty liver disease) and toxins (aflatoxins) [14].

Chronic hepatitis B is the leading cause of new cases of liver cancer worldwide, together with its associated deaths and disability-associated life years (DALYs) [18]. Indeed, in 2015, chronic hepatitis B infection was responsible for 33% of liver-cancer-related mortality worldwide, followed by alcohol (30%), chronic hepatitis C (21%) and other causes (16%). There is, however, significant global variation, with chronic hepatitis B representing the most common cause of liver-cancer-related deaths in areas such as western sub-Saharan Africa (45%) but the least common cause in areas such as central Latin America (8%) [18]. Although hepatitis-B-related HCC is more common amongst individuals with cirrhosis, it may also develop in the absence of advanced liver fibrosis [19]. The oncogenic potential of hepatitis B is multifactorial and important factors include chronic inflammation, HBV DNA integration, dysregulation of cellular pathways by HBx protein and an increase in intra-cellular oxidative stress [20]. Hepatitis B is the prototypic member of the Hepadnaviridae family of viruses, with a relaxed circular double-stranded DNA genome (rcDNA). During reverse transcription of pre-genomic RNA (pgRNA), partially double-stranded DNA is formed 90% of the time. However, use of an alternative DR1 primer results in the formation of an alternative genetic construct called double-stranded linear DNA (dslDNA), which can subsequently integrate into the host genome [21]. It is estimated that this integration event occurs in 1 in 10^5^–10^6^ hepatocytes, and this is postulated to drive HCC by inducing chromosomal instability, the cis-mediated insertional mutagenesis of tumour suppressor and proto-oncogenes, and the expression of mutated HBV genes from the integrated genome [21]. In an illustrative study by Sung et al., they noted that HBV integration was more common in carcinomatous versus adjacent normal liver tissue (86.4% vs. 30.7%) with integration sites occurring at known cancer-related genes including telomerase reverse transcriptase (TERT), mixed lineage leukaemia 4 (MLL4) and cyclin E1 (CCNE1) [22]. Furthermore, integrated HBV DNA has a truncated open reading frame, and its transcription can yield a range of abnormal or truncated proteins, which can in turn induce stress in the endoplasmic reticulum and activate the unfolded protein response [23]. HBx protein may also contribute towards carcinogenesis by interfering with a number of key proto-oncogenic signalling pathways, such as mitogen-activated protein kinase (MAPK) and Wnt/ß- catenin cascades, leading to an increased expression of key proto-oncogenes [24,25]. HBx protein also causes aberrant hypermethylation of CpG islands in key tumour suppressor genes, thus decreasing their transcriptional activity, and has been shown to interfere with the translocation of p53 into the nucleus [26].

The potential benefit of surveillance for HCC amongst individuals chronically infected with hepatitis B has been demonstrated by a number of studies, including a couple of large randomised controlled trials performed in China. In a study performed in the Jiangsu Province between 1989 and 1995 comprising 5581 HBsAg carriers aged between 30 and 69, screening with six monthly alpha-fetoprotein (AFP) resulted in an earlier diagnosis of HCC compared with controls. In their study, the percentage of patients diagnosed with early-stage HCC was 29.6% in the screening group, versus 6.0% in the control group [27]. In another study by Zhang et al., 18,816 people with chronic hepatitis B infection were randomised to a screening programme comprising six monthly ultrasounds and AFP measurement, versus no screening. Despite the low up-take of the screening programme (58.2%), screening resulted in a 37% reduction in overall mortality. Furthermore, screening was associated with a higher proportion of small HCC (45.3% vs. 0%) and surgically resectable tumour detected (46.5% vs. 7.5%), as well as improved survival at 1 year (65.9% vs. 31.2%), 3 years (52.6% vs. 7.2%) and 5 years (46.4% vs. 0%) [28].

In determining the target population for surveillance, it is important to take into consideration the incidence of HCC, the degree of benefit that can be gained from treatment, and the cost-effectiveness of the programme. Expert opinion suggests that HCC surveillance for chronic HBV carriers is cost-effective if the annual incidence is at least 0.2% [29], and this figure has been used to inform international guidelines on HCC screening, including those published by AASLD, EASL and APASL [6,7,8], which are outlined in Table 1. Whilst there is no consensus on a cost-effectiveness threshold below which an intervention would be considered unequivocally cost-effective, the value of USD 50,000 per quality-adjusted life year (QALY) gained is often regarded as a good benchmark [30]. A cost-effective analysis using a Markov model found that twice-yearly ultrasound surveillance for patients with cirrhosis over the age of 50 increased quality-adjusted life expectancy by an average of 8.6 months, but nearly 3.5 years in patients with small, treated tumours. Twice-yearly ultrasound surveillance was associated with an incremental cost-effectiveness ratio of USD 30,700 per QALY gained [31], suggesting that this approach may be reasonably cost effective.

Whilst ultrasound is currently the recommended modality for screening, it has reduced sensitivity in certain populations, such as those with hepatic steatosis or elevated BMI, and is strongly dependent on operator experience [32]. In 2013, Johnson et al. proposed a serum-based tool for estimating the likelihood of the presence of hepatocellular carcinoma in patients with chronic liver disease. The GALAD score was initially developed from a cohort of 670 patients in the United Kingdom, and comprised gender, age and three biochemical markers (alpha fetoprotein, alpha fetoprotein-L3 and des-γ-carboxyprothrombin) [33]. Subsequently, the score has been externally validated in cohorts of patients in Japan, Germany and the USA, with promising results [34,35]. For example, the model provided an area under the receiver operating curve (AUROC) of 0.93 (95% CI 0.92–0.94) and 0.94 (95% CI 0.93–0.96) in the Japanese and German cohorts, respectively. Furthermore, the GALAD score has been shown to be specific, discriminating hepatocellular carcinoma from other hepatobiliary cancers with an AUROC of 0.95 [34], with good discriminative capacity across a range of different aetiologies, including chronic hepatitis B [35]. Other researchers have explored the use of abbreviated magnetic resonance imaging (MRI) as a modality of screening for hepatocellular carcinoma. This method involves the acquisition of a limited number of MRI sequences with the overall aim of reducing the duration of the MRI examination, whilst retaining its diagnostic performance. Over recent years, a range of different approaches have been investigated, including non-contrast protocols combining T2- and diffusion-weighted sequences, dynamic MRI with extracellular contrast, and abbreviated gadoxetate-enhanced hepatobiliary phase imaging [36]. In a recent systematic review comprising 15 studies (3 prospective, 12 retrospective), abbreviated MRI was found to have a higher sensitivity for HCC detection compared with ultrasound (82% vs. 53%), with comparable findings between contrast and non-contrast enhanced MRI [37]. Whilst these results are promising, MRI is contra-indicated in certain groups of patients (e.g., those with magnetic sensitive devices) and there remain concerns about its cost-effectiveness. Furthermore, more studies are needed to define the optimal abbreviated MRI protocol.

## 4. Early Hepatocellular Carcinoma Risk Prediction Scores

Epidemiological studies have identified a number of factors that are associated with increased risk of developing hepatocellular carcinoma (HCC) in those with chronic hepatitis B infection, including the presence of cirrhosis, male gender, higher levels of serum HBV DNA, genotype C infection, specific basal core promoter variations, and a family history of HCC [38,39,40]. Over the last 10–15 years, a number of scores have been developed to model the risk of HCC development more precisely in patients with chronic hepatitis B infection. Indeed, our literature review identified 23 studies that have described the development of an HCC risk prediction model in this group of patients and are of sufficient quality based on the Newcastle–Ottawa Scale. One of the earliest risk prediction scores was GAG-HCC score, developed from a cohort of 820 patients with chronic hepatitis B in Hong Kong. Regression analysis confirmed that increasing age (*p* < 0.001), male gender (*p* = 0.025), higher HBV DNA levels (*p* = 0.02), presence of cirrhosis (*p* < 0.001) and core promoter mutations (*p* = 0.007) were independent risk factors for HCC development. A risk score was derived from these five factors with an area under the receiver operating curve (AUROC) of 0.88 and 0.89 for predicting the development of HCC after 5- and 10-years, respectively. [41]. In 2010, an alternative risk score called CU-HCC was developed based on a cohort of 1005 patients attending the Prince of Wales Hospital in Hong Kong and validated in another cohort of 424 patients. This score comprises five variables, namely, age, albumin, bilirubin, HBV DNA level and absence/presence of cirrhosis, with a possible score ranging from 0 to 44.5. In the validation cohort, the negative predictive values for predicting 5 and 10-year risk of HCC development were 98.3% and 97.3%, respectively [42]. In the following year, another group from South East Asia proposed the REACH-B score, based on a development cohort of 3584 patients in Taiwan and a validation cohort of 1505 patients from three hospitals in Hong Kong and South Korea. A 17-point risk score was developed based on the variables of age, sex, serum alanine aminotransferase, HBeAg status, and serum HBV DNA level. In the validation cohort, the AUROC to predict risk were 0.811 at 3 years, 0.796 at 5 years and 0.769 at 10 years [43].

## 5. Newer Hepatocellular Carcinoma Risk Prediction Scores

A number of the early scores for hepatocellular carcinoma (HCC) prediction were derived from chronic hepatitis B patients who were naïve to antiviral therapy, or only included a small minority of patients receiving antiviral therapy [42]. An important variable in all three of the early risk scores is HBV DNA levels, but in the era of nucleos(t)ide analogues and potent inhibition of viral reverse transcriptase activity, the predictive capacity of HBV DNA levels in treated individuals may be less relevant. The accuracy of GAG-HCC, CU-HCC and REACH-B amongst individuals treated with nucleos(t)ide therapy has subsequently been explored. For example, in a retrospective cohort study of 1531 Asian patients with chronic hepatitis B infection treated with at least 12 months of entecavir therapy, the area under the receiver operating curve (AUROC) of baseline REACH-B, CU-HCC and GAG-HCC scores for predicting HCC development were 0.71 (95% CI 0.62–0.81), 0.80 (95% CI 0.75–0.86) and 0.76 (95% CI 0.70–0.82), respectively [44]. Whilst studies have externally validated the use of these three scores amongst cohorts of Asian patients, their discriminatory performance amongst Caucasian individuals has been found to be relatively modest. In an illustrative, multicentre study of 1666 Caucasian patients receiving entecavir or tenofovir therapy, GAG-HCC, CU-HCC and REACH-B scores were not associated with HCC development in the multivariate analyses and only offered relatively modest predictability [45]. To address this shortcoming, the PAGE-B score was subsequently developed, based on patients’ age, gender and platelets, and developed using a cohort of 1815 Caucasian patients who had received antiviral therapy with nucleos(t)ide therapy for ≥12 months (derivation subset n = 1325, validation subset n = 490). In their derivation datasets, patients with PAGE-B ≤9, 10–17 and ≥18 had 5-year cumulative HCC incidence rates of 0%, 3% and 17%, respectively [46].

The use of PAGE-B in Caucasian populations has been validated by several other research groups. For example, in a study by Riveiro-Barciela et al., the negative predictive value of the low-risk PAGE-B group was re-affirmed, as no patients with a baseline PAGE-B score of ≤ 9 developed HCC over a mean follow-up period of 55 (entecavir) or 49 (tenofovir) months [47]. In another study from the Netherlands, comprising 557 patients of diverse ethnicity, PAGE-B predicted HCC development with good accuracy (c-index of 0.91, 95% CI 0.82–0.99) and was superior to REACH-B (c-index 0.83) and CU-HCC (0.84) scores in this regard [48]. The PAGE-B score has also been validated in a large cohort of Asian individuals, with an AUROC of 0.72 for predicting the 5-year risk of HCC development [49]. The same authors proceeded to propose a modified mPAGE-B score based on age, gender, platelet count and serum albumin levels to improve predictive performance of the model, with an AUROC of mPAGE-B at 5 years of 0.82 [49]. However, in an external validation study of 1330 patients with chronic hepatitis B infection receiving treatment, the addition of albumin provided only a marginal benefit to the performance of the PAGE-B score with Harrell’s c-index of 0.769 for mPAGE-B score and 0.744 for PAGE-B score [50].

More recently, a number of other risk scores have been proposed to predict future risk of HCC development in patients with chronic hepatitis B infection. In 2017, Sohn et al. proposed the HCC-RESCUE model comprising three factors, namely, age, gender and cirrhosis, to predict the risk of HCC development in patients receiving entecavir therapy. Based on their composite score, patients were divided into a low (≤64 points), intermediate (65–84 points) and high-risk group (≥85 points). The AUROC at 1 year, 3 years and 5 years were 0.82, 0.81 and 0.81, respectively [51]. The use of the HCC-RESCUE model has recently been externally validated in a cohort of Caucasian patients by Güzelbulut et al. In their study, AUROCs of the HCC-RESCUE, PAGE-B and mPAGE-B scores to predict HCC risk at 5 years were 0.875, 0.866 and 0.880, respectively. Of note, there was no statistically significant difference in AUROC for the HCC-RESCUE score in patients receiving tenofovir versus entecavir (*p* > 0.05) [52].

In 2017, another HCC risk predictor score called APA-B was proposed for patients receiving entecavir therapy [53]. In their cohort, a total of 1325 patients with chronic hepatitis B were included, with patients randomly assigned to the development (n = 883) and validation (n = 442) groups. The APA-B score was constructed based on age, platelet count and AFP level, with a total score ranging from 0 to 15. In the development group, the AUROCs for predicting HCC risk after 2, 3 and 5 years were 0.877, 0.842 and 0.827, respectively. This score may also have a role in predicting HCC development beyond Year 5 of antiviral therapy. Indeed, in a recent study of 1397 Asian patients receiving entecavir monotherapy, APA-B score at Years 1 and 5 had a higher c-index for the prediction of HCC beyond Year 5 than the corresponding PAGE-B score (*p* = 0.003 and *p* = 0.039, respectively) [54].

For a number of years, it has been appreciated that diabetes is an important risk factor for HCC development in patients with chronic hepatitis B [55]. Based on this, a group of researchers have recently proposed a novel scoring system to predict HCC risk in patients on antiviral therapy called the CAMD score, based on the variables of cirrhosis, age, male sex and diabetes, at the start of treatment initiation. Using a development cohort of 23,851 patients from Taiwan, and validated using a Hong Kong cohort, the c indices for HCC in the development cohort were 0.83 (95% CI 0.81–0.84), 0.82 (95% CI 0.81–0.84) and 0.82 (CI 0.80–0.83) after one, two and three years of therapy, respectively [56]. The CAMD score has subsequently been validated by Kim et al., who followed up 3277 patients with chronic HBV infection in South Korea for a median follow-up of 58.2 months. CAMD scores identified patients who developed HCC with an AUROC of 0.790, compared with an AUROC of 0.769 and 0.760 for mPAGE-B and PAGE-B, respectively. The 5-year cumulative risks of HCC were 1.3% in patients with low CAMD scores (<8), 8.0% in patients with intermediate CAMD scores (8–13) and 24.3% in patients with high CAMD scores (>13) (*p* < 0.001) [57]. Another recently proposed scoring system for HCC risk prediction that includes diabetes is the REAL-B score, based on a total of 8048 patients with chronic hepatitis B infection (derivation set n = 5365, validation set n = 2683) who were receiving antiviral therapy. For this study, patients were recruited from 25 centres across the USA and Asia Pacific region, and the risk score was calculated from seven variables, namely, age, male gender, diabetes, alcohol use, baseline cirrhosis, platelet count and alpha fetoprotein level. Patients are divided into three different risk categories based on their score (0–3 low risk, 4–7 moderate risk, 8–13 high risk), with AUROC for the prediction of a 5-year risk of hepatocellular carcinoma of >0.80 [58].

Finally, attempts have been made to generate a more generic risk model for HCC development that can be applied to patients with different underlying aetiologies of their chronic liver disease. For example, a new score called aMAP has recently been proposed that incorporates a patient’s age and gender, as well as albumin-bilirubin and platelet count [59]. This score was generated from an international collaboration of 17,374 patients with chronic hepatitis. The majority of the study population had chronic hepatitis B infection, of whom 10,578 were of Asian ethnicity and 2510 of Caucasian ethnicity. The remaining patients had chronic hepatitis C infection (3566 individuals) or non-viral hepatitis (720 individuals, the majority of whom had non-alcoholic fatty liver disease). In this large multi-centre study, the aMAP score accurately predicted HCC development, with a c-index significantly higher than other HCC risk scores (*p* < 0.0001 vs. REACH-B, *p* < 0.0001 vs. CU-HCC, *p* = 0.041 vs. PAGE-B, *p* = 0.049 vs. mPAGE-B). The score was validated across a number of cohorts with different ethnicities, and the c-index ranged from 0.82 to 0.87 (95% CI, 0.74–0.97). In the validation cohort, a cut-off aMAP value of 50 was associated with a sensitivity of 85.7–100% and an NPV of 99.3–100% [59].

## 6. Transient Elastography

It is well established that liver cirrhosis is a major risk factor for the development of hepatocellular carcinoma in patients with chronic hepatitis B infection. Over the past few years, there has been validation and increasing use of transient elastography as a non-invasive means of measuring liver stiffness [60], and attempts have been made to incorporate this into novel HCC risk prediction scores. For example, in 2013, Kim et al. proposed a model to predict the three-year risk of hepatocellular carcinoma (HCC) occurrence based on liver stiffness value, age, gender and HBV DNA level. Based on their cohort of 1250 patients recruited from a single tertiary centre, and a median follow-up period of 30.7 months, the risk model offered good discrimination capability, with an area under the receiver operating curve (AUROC) of 0.806 (95% CI 0.738–0.874) [61].

Transient elastography has also been incorporated into the pre-existing CU-HCC and REACH-B scores, to generate newer scores called LSM-HCC and mREACH-B, respectively. Both of these scores have a better predictive performance than their original models. For example, in the initial derivation of the LSM-HCC score comprising liver stiffness measurement, age, serum albumin and HBV DNA levels, the AUROC of LSM-HCC score was noted to be 0.83–0.89, compared with 0.75–0.81 for the CU-HCC score. By applying the cut-off value of 11 (out of a maximum of 30), the LSM-HCC score excluded future HCC with high negative predictive value (99.4–100%) at 5 years [9]. The utility of LSM-HCC score has subsequently been externally validated in a number of studies in the Asian population. For example, in a study of over 1200 patients in South Korea with chronic hepatitis B infection, LSM-HCC strongly predicted HCC risk with an AUROC of 0.809 (95% CI 0.742–0.876), 0.742 (95% CI 0.677–0.809) and 0.765 (95% CI 0.709–0.821) at 3, 5 and 7 years, respectively [62].

The initial REACH-B score was derived from a cohort of 3584 non-cirrhotic patients from the Taiwanese-based REVEAL-HBV study, with a validation cohort of 1505 patients from three hospitals in Hong Kong and South Korea. Variables included in the original REACH-B score included age, sex, serum alanine aminotransferase concentration, HBeAg status and serum HBV DNA levels, to generate a 17-point risk score [43]. In the modified REACH-B model (mREACH-B) based on a cohort of patients treated with antiviral therapy, the serum levels of HBV DNA were substituted for liver stiffness measurement at the stage of complete virological response (defined as HBV DNA < 20 IU/mL). Incorporation of liver stiffness was associated with a better predictive performance compared with the original REACH-B score as determined by the AUROC (0.814 vs. 0.629, respectively) [10]. In an external validation study of over 1200 patients in South Korea, the mREACH-B score was found to have similar or superior predictive capability to PAGE-B and LSM-HCC with an AUROC of 0.824, 0.750 and 0.770 at 3, 5 and 7 years, respectively [62].

## 7. Other Non-Invasive Markers of Liver Fibrosis

Whilst the initial results regarding transient elastography are promising, it is important to note that transient elastography measures liver stiffness as a surrogate marker of fibrosis. Furthermore, a number of other factors can increase liver stiffness, including active hepatic inflammation [63] and heart failure [64]. APRI, consisting of aspartate aminotransferase and platelet count, is a relatively inexpensive and widely available biomarker that has been proposed to predict the degree of liver fibrosis [65]. A recent meta-analysis of 13 studies and 8897 patients has explored the association between APRI index and hepatocellular carcinoma (HCC) risk in patients with chronic viral hepatitis. In the subgroup of patients with chronic hepatitis B infection, an elevated pre-treatment APRI was associated with increased HCC risk (HR 3.16, 95% CI 1.77–5.63) [66]. In another study by Paik at al., the use of APRI, in combination with the fibrosis-4 (FIB-4) score, was used to predict HCC risk in patients with chronic hepatitis B infection and low-level viraemia (defined in their study as < 2000 IU/mL). With a median follow-up of 5.1 years, there were a total of 36 cases of HCC and the 5-year cumulative incidence of HCC was 13.9% (high APRI and FIB-4), 1.4% (high APRI or FIB-4) and 1.2% (low APRI and FIB-4), respectively, suggesting that a combination of non-invasive serum biomarkers could be useful in the risk stratification of this group of patients [67].

Over recent years, research has been conducted to identify and validate serological biomarkers of liver fibrosis, which may facilitate early detection of fibrotic disease. Mac-2 binding protein glycosylation isomer (M2BPGi) is a liver-specific glycosylation isomer of Mac-2 binding protein (M2BP) and has been shown to be a useful serological marker of liver fibrosis in a range of liver conditions, including chronic hepatitis B [68]. Subsequently, a number of studies have explored the potential utility of this novel biomarker in predicting risk of future HCC development. In a study of 1323 patients with chronic hepatitis B, and a median follow-up period of 60.3 months, 52 (3.9%) patients developed HCC. In multivariate analyses, M2BPGi level was an independent predictor of HCC development (HR 1.14, 95% CI 1.14–1.83) [69]. In a study performed by Mak et al. exploring a treatment-naïve population with spontaneous HBeAg seroconversion, the authors demonstrated that median M2BPGi levels at baseline, at 5 years and 10 years after HBeAg seroconversion were significantly higher in patients who developed HCC compared to those who did not (*p* < 0.001). Furthermore, baseline M2BPGi was found to be a significant risk factor predictive of HCC (OR = 4.67, 95% CI 1.30–16.8, *p* = 0.018). The optimal cut-off value was 0.68 COI, which predicted HCC development with 91.7% sensitivity and 80.8% specificity [70].

In the treatment population, levels of M2BPGi have also been shown to predict future risk of HCC. In a study of 899 patients treated with long-term entecavir, baseline serum M2BPGi levels were positively associated with HCC incidence rates (*p* < 0.01) [71]. After categorising the patients into high (≥1.73) and low-level categories (<1.73), baseline M2BPGi ≥1.73 was an independent risk factor for HCC development with an HR of 5.80 (95% CI 3.50–9.60). When comparing the M2BPGi kinetics in patients with or without HCC, the median levels of M2BPGi were consistently higher in the HCC group at a range of time points, including baseline, time of attaining undetectable viral load and end of follow-up/time of HCC diagnosis [71]. In another study of 234 patients treated with entecavir or tenofovir, raised M2BPGi ≥1.215 COI 48 weeks after therapy was an independent risk factor for HCC development with an HR of 5.07 (*p* = 0.004) [72]. In the subgroup of patients who achieve undetectable viral DNA levels with antiviral treatment, Cheung et al. found that higher pre-treatment levels of M2BPGi were demonstrated in the group that subsequently developed HCC versus controls (0.67 vs. 0.41 COI, respectively, *p* < 0.001). With a cut-off value of 0.69, the AUROC of pre-treatment M2BPGi to predict HCC development was 0.70 [73].

## 8. Dynamic Assessment of Long-Term Hepatocellular Carcinoma Risk

Over recent years, a number of studies have explored how the risk of hepatocellular carcinoma (HCC) development may vary over time in those treated with long-term antiviral therapy, and the potential role of transient elastography in this dynamic risk assessment. For example, in a study of 209 patients with chronic hepatitis B infection and advanced fibrosis or cirrhosis, they noted that 67% of patients had achieved sub-cirrhotic range of liver fibrosis after 2 years of antiviral therapy. On multivariate analysis, the achievement of sub-cirrhotic liver stiffness was independently associated with a reduced risk of HCC development (HR 0.485, *p* = 0.047). Patients with a cirrhotic range of liver stiffness after 2 years of treatment were at a higher risk of HCC development than those with sub-cirrhotic liver stiffness (*p* = 0.020) [74]. In another large, multi-centre study of Caucasian patients receiving entecavir or tenofovir treatment, the cumulative HCC incidence rate was 5.7% at 5 years of follow-up (derivation dataset) [46], versus 3.8% between 5 and 12 years [75]. The authors subsequently proposed two risks scores to predict the long-term risk of HCC after 5 years of treatment with anti-viral therapy, both of which incorporate use of transient elastography. The first score, called CAGE-B, uses the variables of age, elastographic evidence of cirrhosis at baseline and Year 5 to calculate a score between 0 and 16. In patients with low (0–5), medium (6–10) and high (11–16) CAGE-B scores, the 12-year cumulative HCC incidence was 0, 0.18 and 1.01 per 100 patient-years, respectively. The second score, called SAGE-B, is based on age and the elastographic presence of cirrhosis at Year 5, regardless of cirrhosis baseline status. In patients with low (0–5), medium (6–10) and high (11–16) SAGE-B scores, the 12-year cumulative HCC incidence was 0, 0.29 and 1.51 per 100 patient-years. Both scores offered 100% NPV for HCC beyond Year 5 in the low-risk groups (scores 0–5) [75].

The authors proposing the CAGE-B and SAGE-B scores recognise the need to prospectively validate the scores further in populations, including those of Asian ethnicity. Indeed, some studies carried out amongst Asian populations have not noticed a significant decrement in HCC risk over time in those treated with antiviral therapy, contrary to the findings of Papatheodoridis et al. [75]. For example, in a recent study of 3156 Asian patients with chronic hepatitis B infection and treated with long-term antiviral therapy, 9.0% developed HCC during the follow-up period. The annual incidence of HCC per 100 person-years during the first 5 years (n = 1671) and after the first 5 years (n = 1485) was similar with no statistically significant difference (1.93% vs. 2.27%, *p* = 0.347) [76]. In another study by Sou et al., the annual incidence of HCC was 2.28% within the first 5 years of treatment, and 1.34% within 5–10 years of therapy. This did not achieve statistical significance (*p* = 0.53), including those with (*p* = 0.85) or without cirrhosis (*p* = 0.47) [54]. The differences noted between the studies with regard to HCC risk may be secondary to key demographic differences and the viral genotypes that are predominant in their respective populations. Table 2 summarises some of the recent HCC risk prediction models that have been developed.

## 9. Hepatitis B Core-Related Antigen (HBcrAg)

A number of studies have shown that treatment with potent nucleos(t)ide therapy can reduce, but not eliminate the risk of hepatocellular carcinoma, and this may be secondary to non-modifiable risk factors (such as male gender, age, HBV genotype) or carcinogenic events that take place before treatment initiation (such as HBV integration) [38]. Over recent years, several studies have explored how novel viral biomarkers can inform and refine hepatocellular carcinoma (HCC) risk prediction scores. HBV cccDNA is the key molecule responsible for the persistence of the virus within infected hepatocytes, acting as a stable, extra-chromosomal template for the transcription of all HBV viral transcripts [77]. Importantly, the level of cccDNA transcription has been shown to correlate directly with disease progression and clinical outcomes [78], but direct assessment of the cccDNA reservoir is limited in clinical practice by the need for a liver biopsy. Among the available biomarkers, HBV DNA correlates with intrahepatic cccDNA levels in untreated patients, but the potent inhibition of HBV polymerase by nucleos(t)ide analogues results in a rapid fall in HBV DNA levels during treatment [79], rendering this test less informative for predicting HCC risk in a large group of patients.

Hepatitis B core-related antigen (HBcrAg) is a biomarker that simultaneously measures three proteins encoded by the precore/core gene, namely, hepatitis B core antigen (HBcAg), hepatitis B e antigen (HBeAg) and a 22 kDa core-related protein (p22cr) that forms the capsid of HBV DNA negative Dane-like particles [80]. All three proteins share an identical 149 amino acid sequence and can be measured by modern immunoassays [81]. Levels of HBcrAg have been shown to correlate with cccDNA levels and transcriptional activity in both treated and untreated individuals with chronic hepatitis B infection. In a recent meta-analysis of 14 studies (1271 patients) comprising a mixture of treated and untreated patients with chronic hepatitis B infection, there was a high correlation between serum HBcrAg and intrahepatic HBV cccDNA levels (r = 0.641, 95% CI 0.51–0.74, *p* < 0.001) [82]. Further subgroup analysis found that the correlation between HBcrAg and intrahepatic HBV cccDNA was high in both the HBeAg-positive (r = 0.678, 95% CI 0.40–0.84, *p* < 0.001) and HBeAg-negative patients (r = 0.578, 95% CI 0.34–0.74, *p* < 0.001). Finally, in a direct, head-to-head comparison, the authors found the performance of HBcrAg to correlate more strongly with HBV cccDNA levels, compared with HBsAg (r = 0.665 vs. r = 0.475, *p* < 0.001) [82]. In addition to correlating with intrahepatic cccDNA levels, HBcrAg has also been shown to correlate with cccDNA transcriptional activity as defined by the pgRNA:cccDNA ratio (r = 0.52, *p* < 0.0001) [83].

Having been established as a good surrogate marker of cccDNA levels and transcriptional activity, several research groups have explored the potential use of HBcrAg in the prediction of HCC development. In a study of 1031 untreated patients with chronic hepatitis B, the authors found that elevation of HBcrAg > 2.9 logU/mL was independently associated with the incidence of HCC (hazard ratio 5.05, 95% CI 2.40–10.63) and superior to HBV DNA in terms of predictive power for HCC development. Indeed, the AUROCs for predicting 2-year and 5-year risk of HCC were 0.80 and 0.68 (HBcrAg) vs. 0.75 and 0.63 (HBV DNA) [84]. Levels of HBcrAg may be especially useful in identifying a subgroup of patients with intermediate viral load and increased risk of hepatocellular carcinoma. In a study by Tseng et al., comprising 2666 patients and a mean follow-up of 15.95 years, HBcrAg levels of 10 KU/mL or more identified a subgroup of HBeAg-negative disease and an intermediate viral load (HBV DNA levels 2000 to 19 999 IU/mL) who were at increased risk of HCC (hazard ratio 6.29, CI 2.27–17.48) [85]. Furthermore, in a recent study of 1400 patients, serum HBcrAg level above 2.9 log U/mL at the time of starting antiviral therapy was an independent risk factor for HCC development in HBeAg-negative patients by multivariate analysis (HR 2.13, 95% CI 1.10–4.14, *p* = 0.025). In the same study, HBsAg level did not show any correlation with the risk of HCC in any subgroups [86].

Whilst nucleos(t)ide analogues can potently reduce the levels of HBV DNA, it has a more modest effect on the levels of cccDNA and HBcrAg. As an example, in a study of 39 patients treated with either entecavir or lamivudine, 65% of individuals with undetectable HBV DNA (<300 copies/mL) still had detectable HBcrAg at the end of treatment [87]. In a study of 222 patients on continuous entecavir treatment and followed up for 7 years, the median rate of decline was 0.244 log kU/mL/year with 32.0% of patients demonstrating undetectable HBcrAg levels at Year 7 [88]. In treatment-experienced patients, a number of studies have shown that HBcrAg levels correlate with risk of HCC development. In a retrospective cohort study of 1268 patients treated with nucleos(t)ide analogues, Hosaka et al. found that individuals with persistently high on-treatment HBcrAg levels at one year had a greater risk of HCC development compared to those with low HBcrAg levels, with significant findings in both the HBeAg-positive (HR 6.15, 95% CI 1.89–20.0, *p* = 0.003) and HBeAg-negative cohorts (HR 2.54, 95% CI 1.40–4.60, *p* = 0.002) [89]. In a cohort of entecavir-treated chronic hepatitis B patients with a longer follow-up, Mak et al. found that serum HBcrAg levels were persistently higher in the HCC group compared to match controls at baseline (*p* = 0.025), 3 years (*p* = 0.007) and 5 years (*p* = 0.009) of entecavir treatment, after adjustment for other covariates including age, gender, baseline HBV DNA and cirrhosis status [11]. In patients who achieve HBV DNA disappearance with nucleos(t)ide analogue therapy but subsequently develop HCC, Ando et al. noted a 1-year, 3-year and 5-year cumulative incidence of HCC of 0.0%, 13.6% and 17.7%, respectively, in patients with serum HBcrAg levels >3.4 log U/mL at the time of HBV DNA disappearance, and 0.0%, 0.0% and 2.4%, respectively, in patients with HBcrAg levels <3.4 log U/mL [12]. Increased levels of HBcrAg post-nucleos(t)ide analogue therapy is also associated with increased risk of HCC development, with a cut-off value of ≥7.8 kU/mL yielding an AUROC of 0.61 and an OR of HCC development of 3.27 in a study by Cheung et al. Amongst the non-cirrhotic patients included in their study, the median values of post-treatment HBcrAg level in the HCC group and controls were 10.2 and 1.0 kU/mL, respectively (*p* = 0.001) [90].

## 10. Discussions

Chronic hepatitis B remains a global health problem, with the World Health Organisation setting a target of reducing hepatitis-related mortality by 65% by the year 2030 [91]. Despite the widespread use of nucleos(t)ide analogue therapy, hepatocellular carcinoma remains a major cause of morbidity and mortality in this group of patients [18]. A number of models have been developed to define this risk and are typically based on a combination of non-modifiable host risk factors (age, male gender), co-morbidities (diabetes), biochemical parameters (albumin, bilirubin, alanine aminotransferase, platelet count), viral markers (HBV DNA level, basal core promoter mutations) and the degree of liver fibrosis/cirrhosis. Importantly, the first three scores (GAG-HCC, CU-HCC, REACH-B) were developed in untreated Asian patients with chronic hepatitis B infection [41,42,43] and have shown relatively poor predictive performance in treated Caucasian populations [45]. Furthermore, many of the newer scores developed for treated Asian patients (APA-B, CAMD) have not yet undergone external validation in non-Asian populations [53,56]. PAGE-B score was specifically developed in a cohort of treated Caucasian patients and has undergone external validation in other Caucasian and Asian populations, with promising results [47,48,49]. The use of these risk scores in sub-Saharan Africa has not been extensively studied, and no study has externally validated all proposed HCC risk scores in the same cohort of patients, making it difficult to make direct comparisons. This is particularly important as hepatitis B is endemic in sub-Saharan Africa with an estimated seroprevalence of 6.1% (95% CI 4.6–8.5) [92] and accounts for approximately 87,890 deaths in this region each year [92]. In addition, the age-standardised incidence of hepatocellular carcinoma in sub-Saharan Africa is as high as 41.2 per 100,000 people per year, with chronic hepatitis B representing the most common underlying aetiology [93]. Once diagnosed with hepatocellular carcinoma, the prognosis is particularly poor in these countries with an estimated 93% of individuals dying within one year of onset of symptoms [93]. Finally, co-infection with HIV adds further clinical burden in this region with approximately 2.6 million HIV-HBV co-infected individuals in sub-Saharan Africa, and they typically display a more aggressive phenotype, including higher levels of viraemia and progression to cirrhosis and hepatocellular carcinoma [94].

It is important to recognise that the risk of hepatocellular carcinoma (HCC) development in patients with chronic hepatitis B infection may be influenced by host factors including co-morbidities (such as diabetes), and certain behaviours (such as excessive alcohol intake). Indeed, the recently proposed REAL-B score incorporated both diabetes and alcohol use into their risk prediction model and showed good discriminative capability with an AUROC of >0.80 for HCC risk prediction at 3, 5 and 10 years [58]. With the increasing prevalence of non-alcoholic fatty liver disease worldwide [95,96], it is important that future risk models consider these important risk factors. In addition, patients with chronic hepatitis B and co-infection with HIV and/or hepatitis C have often been excluded from the derivation and/or validation cohorts for the risk prediction models, and this subgroup of patients are not currently well represented in the HCC risk scores.

Many of the early risk scores, such as GAG-HCC, CU-HCC and REACH-B, were developed in untreated Asian patients with chronic hepatitis B infection. In the era of nucleos(t)ide analogue therapy, the discriminatory value of HBV DNA level may be less useful, given the potent inhibitory effect of these medications on reverse transcriptase activity [79]. Levels of cccDNA correlate closely with disease progression and clinical outcomes [78], but direct assessment of the cccDNA reservoir is limited in clinical practice by the need for a liver biopsy. Recently, several research groups have explored the potential use of surrogate markers of cccDNA levels, such as hepatitis B core-related antigen, in the prediction of HCC risk. Early studies have been promising and shown levels of HBcrAg to be superior to DNA levels in predicting the HCC risk of both treatment-naïve and recipient patients, as determined by the AUROC [84]. Further research is needed to externally validate the role of HBcrAg in predicting HCC risk in other populations, including Caucasian groups and those residing in sub-Saharan Africa.

## 11. Conclusions

In conclusion, hepatocellular carcinoma remains a significant cause of morbidity and mortality in patients with chronic hepatitis B, despite the use of nucleos(t)ide analogues. Over the past two decades, a number of different risk prediction models have been developed and further studies are needed to externally validate these models amongst different populations, especially those living in sub-Saharan Africa. In the future, novel surrogate markers of cccDNA transcriptional activity, as well as improved non-invasive markers of liver fibrosis, may lead to refinement of the risk scores. Finally, with the expected increase in obesity and non-alcoholic fatty liver disease over the next couple of decades, new risk scores should reflect the interplay between chronic hepatitis B and host risk factors in determining overall HCC risk.

## Figures and Tables

**Table 1:** Comparison of international guidelines for HCC surveillance in patients with chronic hepatitis B infection.

	AASLD (2018) [7]	EASL (2018) [6]	APASL (2017) [8]
**Screening** **population**	All HBsAg positive patients with: Cirrhosis Asian or black men over 40 Asian women over 50 1st degree relative with history of HCC Co-infection with hepatitis D infection	Cirrhotic patients, Child Pugh A and B Cirrhotic patients, Child Pugh C, awaiting liver transplant Non-cirrhotic HBV patients at intermediate or high risk of HCC (PAGE-B score of 10–17 and ≥18 points, respectively, Caucasian patients) Non-cirrhotic F3 patients, based on individual risk assessment	Cirrhotic patientsNon-cirrhotic HBsAg positive patients if: Asian females aged > 50 years Asian males aged > 40 years African patients aged > 20 years History of HCC in the family
**Modality of screening**	Ultrasound every 6 monthsInsufficient evidence for or against addition of AFP monitoring	Ultrasound every 6 months No recommendation can be made about utility of AFP for early tumour detection when used to complement ultrasound surveillance	Combination of ultrasound and serum AFP every 6 months Measurement of AFP alone is not recommended for routine surveillance of HCC
**Other** **comments**	Advises against screening patients with cirrhosis and Child Pugh C, unless they are on the transplant waiting list	Acknowledges lack of a universal prognostic model to assess risk of developing HCC	Surveillance strategies in those with decompensated Child Pugh C cirrhosis may not be cost-effective, unless they are awaiting liver transplantation

**Table 2:** Summary of recently developed HCC risk prediction models.

	mREACH-B [10]	mPAGE-B [49]	HCC-RESCUE [51]	APA-B [53]	CAMD [56]
**Patients, n**	192	3001	2061	1325	23,851 (Development cohort) 19,321 (Validation cohort)
**Population**	South Korea	South Korea	South Korea	Taiwan	Taiwan, Hong Kong
**% cirrhosis**	46.9%	19.1% (Development cohort) 20.1% (Validation cohort)	39% (Development cohort) 35% (Validation cohort)	36.3%	26.45 % (Development cohort) 7.10% (Validation cohort)
**Risk score** **parameters**	Age, sex, ALT, HBeAg, liver stiffness	Age, gender, platelet count, serum albumin	Age, sex, cirrhosis	Age, platelet count, AFP level	Cirrhosis, age, sex, diabetes mellitus
**AUROC for HCC development**	0.814 at 3 years	0.82 at 5 years (Validation cohort)	0.768 at 5 years (Development cohort) 0.809 at 5 years (Validation cohort)	0.827 at 5 years (Development cohort) 0.862 at 5 years (Validation cohort)	0.82 at 3 years (Development cohort) 0.75 at 3 years (Validation cohort)
**Interpretation of results**		Scores range from 0 to 21. Low risk (≤8 points), intermediate (9–12), high risk (≥13 points)	Scores range from 18 to 113. Low risk (≤64 points), intermediate risk (65–84), high risk (≥85)	Scores range from 0 to 15. Low risk (0–5), medium risk (6–9), high risk (10–15)	Scores range from 0 to 19. Low risk (<8 points), intermediate risk (8–13 points) and high risk (>13 points)

## Data Availability

Not applicable.
